# Transcriptomic analysis of rapeseed (*Brassica napus*. L.) seed development in Xiangride, Qinghai Plateau, reveals how its special eco-environment results in high yield in high-altitude areas

**DOI:** 10.3389/fpls.2022.927418

**Published:** 2022-08-02

**Authors:** Huiyan Xiong, Ruisheng Wang, Xianqing Jia, Hezhe Sun, Ruijun Duan

**Affiliations:** ^1^College of Agriculture and Animal Husbandry, Qinghai University, Xining, China; ^2^Academy of Agricultural and Forestry Sciences of Qinghai University, Key Laboratory of Spring Rape Genetic Improvement of Qinghai Province, Rapeseed Research and Development Center of Qinghai Province, Xining, China; ^3^Key Laboratory of Plant Nutrition and Fertilizer, Ministry of Agriculture and Rural Affairs, Institute of Agricultural Resources and Regional Planning, Chinese Academy of Agricultural Sciences (CAAS), Beijing, China; ^4^College of Eco-Environmental Engineering, Qinghai University, Xining, China

**Keywords:** rapeseed (*Brassica napus* L.), special environment, plateau, daylight length, transcriptome analysis, seed development

## Abstract

As one of the most important oil crops, rapeseed (*Brassica napus*) is cultivated worldwide to produce vegetable oil, animal feed, and biodiesel. As the population grows and the need for renewable energy increases, the breeding and cultivation of high-yield rapeseed varieties have become top priorities. The formation of a high rapeseed yield is so complex because it is influenced not only by genetic mechanisms but also by many environmental conditions, such as climatic conditions and different farming practices. Interestingly, many high-yield areas are located in special eco-environments, for example, in the high-altitude Xiangride area of the Qinghai Plateau. However, the molecular mechanisms underlying the formation of high yields in such a special eco-environment area remain largely unknown. Here, we conducted field yield analysis and transcriptome analysis in the Xiangride area. Compared with the yield and environmental factors in the Xinning area (a low-yielding area), we found that the relatively longer daylight length is the key to high rapeseed yield in the Xiangride area, which leads up to a 52.1% increase in rapeseed yield, especially the increase in thousand seed weight and silique number (SN). Combined with transcriptome H-cluster analysis and Gene Ontology (GO) and Kyoto Encyclopedia of Genes and Genomes (KEGG) functional analyses, we can assume that the grain development of rapeseed in the Xiangride area is ahead of schedule and lasts for a long time, leading to the high-yield results in the Xiangride area, confirmed by the expression analysis by quantitative real-time polymerase chain reaction (qRT-PCR) of yield-related genes. Our results provide valuable information for further exploring the molecular mechanism underlying high yield in special ecological environments and provide a helpful reference for studying seed development characteristics in special-producing regions for *Brassica napus*.

## Introduction

Rapeseed (*Brassica napus*) is one of the most important oil crops and is cultivated worldwide to produce vegetable oil, animal feed, biodiesel, etc. (Chalhoub et al., 2014; Mason and Snowdon, 2016; Hu et al., 2021). With rapid increases in population and the need for renewable energy, the demand for rapeseed also continually rises. Therefore, the breeding and cultivation of high-yield rapeseed varieties have become top priorities (Slafer, 2003; Hu et al., 2009; Khan et al., 2021). Seed yield can be improved by taking the direct component traits and the other indirect contributing traits into consideration. For the representative oil crop rapeseed, high yield is determined mainly by three yield component traits: silique number (SN), seed number per silique (SPS), and seed weight/thousand seed weight (SW/TSW). SN and SPS determine the total number of seeds per plant, and SW/TSW has the most significant effect on seed yield (Leon, 1993; Diepenbrock, 2000; Shi et al., 2009; Ding et al., 2012; Luo et al., 2015, 2017). High rapeseed yield is also closely associated with many yield-related traits, such as plant height (PH) and silique length (SL). In addition to being controlled by yield-related genes, an increasing number of studies have shown that these traits are also highly influenced by environmental conditions (Quijada et al., 2006; Udall et al., 2006; Chen et al., 2007; Li et al., 2014; Fu et al., 2015; Zhao et al., 2016, 2021; Zheng et al., 2017; Raboanatahiry et al., 2018).

The effects of the environmental factors on all growth and development processes occur throughout the whole plant life cycle (Quarrie et al., 2006). For the same cultivars, various planting environments lead to different yield potentials, and some special eco-environments can lead to high yields, for example, Taoyuan Township, Yunnan Province and Xiangride area, Qaidam Basin, Qinghai Province in China (Ying et al., 1998a,b; Katsura et al., 2008). The Qaidam Basin, a vast intermountain basin in the northwest Qinghai Province, Qinghai-Tibetan Plateau, is also the largest basin in China with the highest elevation. The agricultural distribution area of the Qaidam Basin has sparse precipitation, a dry climate, low relative humidity, low moisture content in the air, low humidity, and high atmospheric transparency. Significantly, the annual sunshine hours exceed 3,000, and the yearly total solar radiation is approximately 70. The Xiangride area in the Qaidam Basin has become one of the typical high-yielding areas of crops in China due to its special geographical and ecological environment (Su and Pan, 1981; Shi et al., 2014). However, the genetic and molecular mechanisms underlying the high yield formation of crops in this special high-yield Xiangride area are still unclear.

Seed development is an important stage in determining the yield of rapeseed (Hay and Schwender, 2011; Basnet et al., 2013; Borisjuk et al., 2013; Liu et al., 2015a; Gupta et al., 2017). A previous study suggested that cellular activities during seed filling in *B. napus* begin with sugar mobilization, followed by sequential surges in amino acid, lipid, and storage protein synthesis (Hajduch et al., 2006). Based on three stages in seed filling, they divided the expression trends into different functional stages. The first stage includes proteins expressed mainly at the early stages of seed filling. These proteins are involved in glycolysis, respiration, metabolism of sugars, signal transduction, metabolism of amino acids, proteolysis, and defense. Proteins of the second stage, involved in photosynthesis and lipid metabolism, exhibited the highest expression at the midpoint of seed filling. Finally, detoxification, seed maturation, and seed storage proteins are highly abundant at the end stage of seed filling (Hajduch et al., 2006; Huang et al., 2013). Seed development is highly affected by environmental factors, such as sunshine duration, temperature, and water. Thus, we also proposed that the special eco-environment in the Xiangride area could affect the seed development stages and further cause the high yield of rapeseed.

In this study, we performed yield component analysis and comparative transcriptomic analysis of seed development of Qingza 5, a spring rapeseed variety, in different environments to determine the primary yield component responsible for differences in high-yield environments. The differentially expressed genes (DEGs) related to high yield were analyzed, and the high-yield-related genes were also explored by RNA-Seq and quantitative real-time polymerase chain reaction (qRT-PCR). The research results will help to better understand the molecular mechanism of high yield formation in the Xiangride area of the Qaidam Basin, and have important theoretical and practical significance for spring rapeseed breeding and improving yield potential.

## Materials and methods

### Plant materials and growth conditions

Field experiments were conducted in 2015 in the Xining area (XN, 101°49′17″E, 36°34′3″N, altitude 2,320 m) and Xiangride area (XRD, 97°48′9.68″E, 36°4′13.68″N, altitude 2,997 m), Qinghai Province, China, during the rapeseed growing season from April to August. The average annual temperature and rainfall of the XN area were 6.4°C and 306.2 mm, respectively, and those of the XRD area were 4.2°C and 142.5 mm, respectively (for details, see Additional File 1: Supplementary Table 1). A widely planted rapeseed (*B. napus*) variety, Qingza 5, was selected and planted at the experimental farm of Qinghai University (Xining, Qinghai Province, China) and Xiangride farm (Dulan, Qinghai Province, China). The field experiment followed a randomized complete block design with three replicates for every 20 plots. Each plot had an acreage of 12 m^2^ and consisted of 200 plants. The planting density was 20 cm between plants within each row and 30 cm between rows. Field management followed regular planting practices, and the planting practices in the two places were identical. At the grain filling stage, three replicates of siliques from 30 or 40 days after flowering were collected for RNA extraction. All seeds were gathered, snap-frozen in liquid nitrogen, and kept at −80°C for further use.

### Measurement of rapeseed yield traits

Measurements of yield per plant, SN, thousand seed weight (TSW), and SPS were performed after harvest. Ten plants growing uniformly in three replications from each plot in the producing area were chosen for trait evaluation. TSW was calculated based on the average weight of 1,000 fully developed open-pollinated dry seeds.

### Collection of climate information

General climate data of XN and XRD in 2015, including average temperature, rainfall, daylight length, and average soil temperature (of 20 cm), were obtained from the China Meteorological Administration.^1^

### RNA extraction, RNA-seq library construction, sequencing, and data analysis

Total RNA was isolated by using the TRIzol kit (Invitrogen, Carlsbad, CA, United States) and purified using an mRNA purification kit (Promega, Shanghai, China) following the manufacturer’s instructions with some modifications. RNA degradation and contamination were monitored on 1% agarose gels and treated with RNase-free DNase I (Thermo Fisher Scientific, Waltham, MA, United States) to remove any contaminating DNA. The quality and integrity of the extracted RNAs were assessed using the NanoPhotometer^®^ spectrophotometer (Implen, Westlake Village, CA, United States) and the RNA Nano 6000 Assay Kit of the Bioanalyzer 2100 system (Agilent Technologies, Santa Clara, CA, United States). All these RNA samples should display R260/280 at 1.8–2.0, and the threshold of the RNA integrity number (RIN) was set to at least 8. After the quality assessment, 3 μg of RNA per sample was further processed by the purification of polyA-containing mRNA, mRNA fragmentation, double-stranded cDNA synthesis, and polymerase chain reaction (PCR) amplification, and RNA-Seq libraries were generated using the NEBNext^®^ Ultra RNA Library Prep Kit for Illumina^®^ (NEB, Ipswich, United Kingdom) following the manufacturer’s instructions. The final cDNA libraries were sequenced on an Illumina HiSeq 2000 platform by the BGI Tech Solutions Co., Ltd. (BGI-Tech, Shenzhen, Guangdong, China). To preferentially select cDNA fragments of preferentially 150–200 bp in length, the library fragments were purified with the AMPure XP system (Beckman Coulter, Brea, CA, United States). At the same time, the Q20, Q30, and GC contents of the clean data were calculated.

Rapeseeds “*Darmor-bzh*” reference genome and gene annotation files were downloaded from the genome website BRAD (http://brassicadb.cn/). The index of the reference genome was built using Bowtie v2.2.3, and paired-end clean reads were aligned to the reference genome using TopHat v2.0.12. HTSeq v0.6.1 was used to count the read numbers mapped to each gene. Then, the fragments per kilobase of transcript per million mapped reads (FPKM) value of each gene was calculated based on the length of the gene and read count mapped to this gene. Differential expression analysis of the two conditions/groups was performed using the DESeq R package (1.18.0). The resulting *P-*values were adjusted using Benjamini and Hochberg’s approach for controlling the false discovery rate. Genes with an adjusted *P*-value <0.05 found by DESeq were considered differentially expressed genes. Gene Ontology (GO) enrichment analysis of differentially expressed genes (DEGs) was implemented by the GOseq R package, in which gene length bias was corrected. GO terms with corrected *P* values less than 0.05 were considered significantly enriched DEGs. KOBAS software was used to test the statistical enrichment of DEGs in Kyoto Encyclopedia of Genes and Genomes (KEGG) pathways.

### Quantitative real-time PCR analysis

The transcript levels of 11 candidate DEGs of yield-related genes were verified by quantitative real-time PCR (qRT-PCR). Total RNA was treated with DNase, and first-strand cDNA was generated using an AMV First Strand cDNA Synthesis Kit (Sangon, Shanghai, China). SYBR-based qRT-PCRs (SYBR Green I, ABI, Bel Air, MD, United States) were performed on a LightCycler 480 system (Roche, Basel, Switzerland) using the following reaction conditions: 95°C for 3 min followed by 40 cycles of 95°C for 15 s and 60°C for 40 s. The actin gene was used as the internal standard. Three independent biological and technological replicates were performed. The relative transcription level was calculated according to the 2^–ΔΔCt^ method with actin reference genes as a control. Primers are available in Supplementary Table 2.

## Results

### Comparison of yield and its contributing traits between two different areas

**FIGURE 1:**
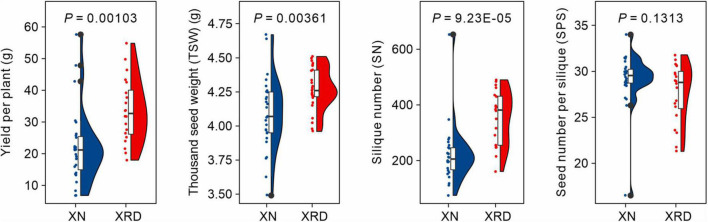
Comparisons of yield-related traits of rapeseed plants in XN and XRD areas.

To evaluate the differences in rapeseed yield among different producing areas and uncover the effects of unique environmental factors on rapeseed yield, all field experiments in this study were conducted in the Xining area (XN) and Xiangride area (XRD) during the rapeseed growing season from April to August in 2015. XRD is a representative high-altitude and high-yield producing area in the Qinghai Plateau. After harvest, we compared the yield of rapeseed in these two different regions and found that, as expected, the yield per plant was significantly higher in the XRD area. To dissect yield traits, we then analyzed the three main factors of yield formation, including SN, thousand seed weight (TSW), and SPS. The results showed no difference in SPS between the two areas, but the SN and TSW of rapeseed in the XRD area were significantly greater than the SN and SW of rapeseed in the XN area (Figure 1 and Supplementary Table 3). Specifically, the SN and yield per rapeseed plant in the XRD area were 59.6 and 52.1% higher than the SN and yield per rapeseed plant in the XN area.

**FIGURE 2:**
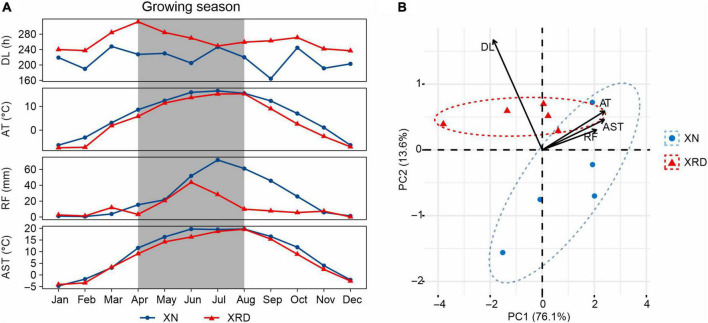
Comparison of the environmental factors of the XN and XRD areas. **(A)** General climate information of XN and XRD areas in 2015. Months with the gray background are the growing season of rapeseed (April to August). AT, average temperature; RF, rainfall; DL, daylight length; AST, average soil temperature at 20 cm. All data were obtained from the China Meteorological Administration and are listed in Supplementary Table 1. **(B)** Principal component analysis of average temperature, rainfall, daylight length, and average soil temperature of XN and XRD in the growing season. Together, these two axes explain a robust 89.7% of the overall variation.

To reveal the main difference in environmental factors between the two growth areas, we compared the average temperature (AT), rainfall (RF), daylight length (DL), and average soil temperature of 20 cm (AST) recorded in the planting season in 2015 (Figure 2A). The AT, RF, and AST were slightly different, but DL was significantly different between the XN and XRD areas. At the same time, compared with XN, DL is the only factor with better data in the XRD area (Supplementary Table 1). To assess the relative contributions of these environmental factors, we performed a principal component analysis (PCA) (Figure 2B). The top two principal components combined to explain a robust 89.7% of the overall variation and have a high degree of interpretation of all environmental factors (Supplementary Table 1 and Supplementary Figure 1). The results showed that DL is the main factor contributing to the environmental difference between the XN and XRD areas. Taken together, these results indicate that the higher TSW and SN were associated with longer daylight length in the XRD area.

### Transcriptome sequencing

In *Brassica napus*, 30–40 days after flowering was the fastest-growing period for seed development, which determined mainly yield formation (Basnet et al., 2013; Liu et al., 2015a). To understand the possible mechanisms of the molecular regulation of higher yields in the special XRD eco-environment, we conducted a comparative transcriptomic study of rapeseed at 30 and 40 days after flowering (DAF) in two different areas (XRD and XN) with three biological replicates. In total, 210.4 million short reads were generated, with 203.9 million high-quality clean reads selected for further analysis. After trimming the adaptor sequences and removing low-quality and short reads, a total of 7.94 G, 7.42 G, 7.98 G, and 7.19 G nucleotides were generated from the XN1 (Xining area, 30 DAF), XN2 (Xining area, 40 DAF), XRD1 (Xiangride area, 30 DAF), and XRD2 (Xiangride area, 40 DAF) libraries, respectively. The minimum Q30 was more than 93%, and the average GC (guanine-cytosine) content was 47.69%, suggesting that the sequencing data were highly accurate and reliable. Then, the total reads of XN1 (53,050,694), XN2 (49,526,474), XRD1 (53,324,798), and XRD2 (48,055,842) were aligned with the reference genome. On average, 76.6%, 81.04%, 80.63%, and 81.14% of the reads were successfully mapped to the reference genome. By comparison with the reference rapeseed genome, all 203.9 million clean reads were assembled into 104,695 genes using Cufflinks, providing massive data for further analysis. Detailed information on quality control and distribution is shown in Supplementary Tables 4, 5. These results suggested that the RNA-Seq data used in the present study were highly reliable.

### Identification of differentially expressed genes

**FIGURE 3:**
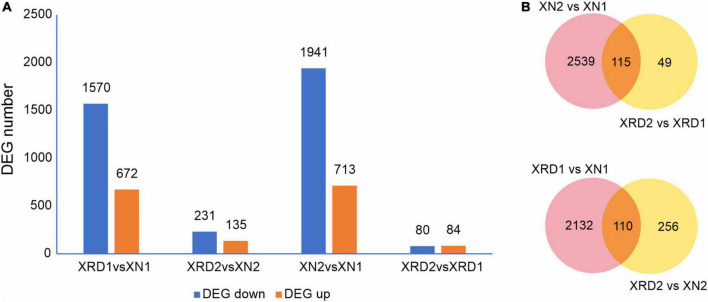
DEGs in different regions at different time points. **(A)** Numbers of upregulated and downregulated DEGs in XN and XRD at different time points. **(B)** Venn diagram of DEGs in different regions at different time points.

To identify the differentially expressed genes (DEGs) between the XN and XRD samples that had relatively high abundance, the fragments per kilobase of exon per million mapped reads (FPKM) value was used to normalize the gene expression levels. The DEGs were defined as the fold change of the normalized reads per kilobase of transcript per million mapped reads (RPKM) at log2Ratio ≥1 and *q* value ≤0.005. Their detailed expression information is shown in Supplementary Table 6. Compared with transcriptomic data from XN and XRD areas, the results showed that a total of 2,242 genes were differentially expressed between the two regions at 30 DAF, of which 1,570 genes were downregulated, and 672 genes were upregulated in rapeseeds planted in XRD compared with the rapeseeds planted in the XN region. A total of 366 genes were differentially expressed at 40 days between rapeseeds planted in XN and XRD, of which 231 genes and 135 genes were downregulated and upregulated in rapeseeds planted in XRD, respectively. There were also 2,654 DEGs identified in rapeseeds planted in the XN area at different times, of which 1,941 genes and 713 genes were downregulated and upregulated at 40 days compared with 30 days, respectively. In addition, 164 genes were differentially expressed in rapeseeds planted in the XRD area at different times, of which 80 and 84 genes were downregulated and upregulated, respectively (Figure 3A).

To further study the effects of different time points and regions on rapeseed yield, we carried out a Venn diagram of the DEGs (Figure 3B and Supplementary Table 7). The results showed 115 common DEGs at different times, representing the changes in rapeseed seed development from 30 to 40 days. Among these DEGs, the BnaC07g42500D (xyloglucan endotransglucosylase/hydrolase protein 24, XTH24) gene was downregulated by more than 100 times in the XN region. In contrast, in the XRD region, this gene was downregulated by more than ten times, indicating that this gene may control the size of the pod by controlling the cell wall decomposition at 30 days. The later disappearance indicated that the pod was transferred into the material accumulation process. The BnaC05g31880D (vacuolar-processing enzyme delta-isozyme) gene has similar trends and may play a similar role in the rapeseed development stage. Most of the upregulated DEGs were dramatically changed in the XN region compared to the downregulated DEGs, but the changes in the XRD region were relatively small, except for BnaC06g13400D and BnaA07g15220D. These two genes are omega-hydroxypalmitate O-feruloyl transferases, which may be involved in the synthesis of aromatics of the suberin polymer, specifically affecting the accumulation of the ferulate constituent of suberin in roots and seeds. The 49 DEGs in Figure 2B were unique to the seed development of the special eco-environment XRD region, including 34 upregulated DEGs and 15 downregulated DEGs. Some regulatory genes were found in the unique development-related genes in the XRD area, including auxin-repressed 12.5 kDa protein involved in plant hormone regulatory pathways, adenosine triphosphate (ATP) synthetase involved in energy conversion and substance transfer transporter (organic cation/carnitine transporter), protein kinases (receptor-like serine/threonine-protein kinase), lipid degrading enzyme (probable peroxygenase 3), and superoxide dismutase (SOD) involved in later oxidation, degradation, etc. When DEGs in different regions were merged, 110 DEGs in Figure 2B may represent that these genes were related to the differences between the two areas, which can be considered as regional difference DEGs. In these DEGs, circadian clock-related genes (Protein LHY, CCA1), hormone-related genes (Protein EXORDIUM), and other regulatory proteins (TAR1_KLULA Protein), as well as lipid-related enzymes (Delta-9 acyl-lipid desaturase), were common regional DEG.

**FIGURE 4:**
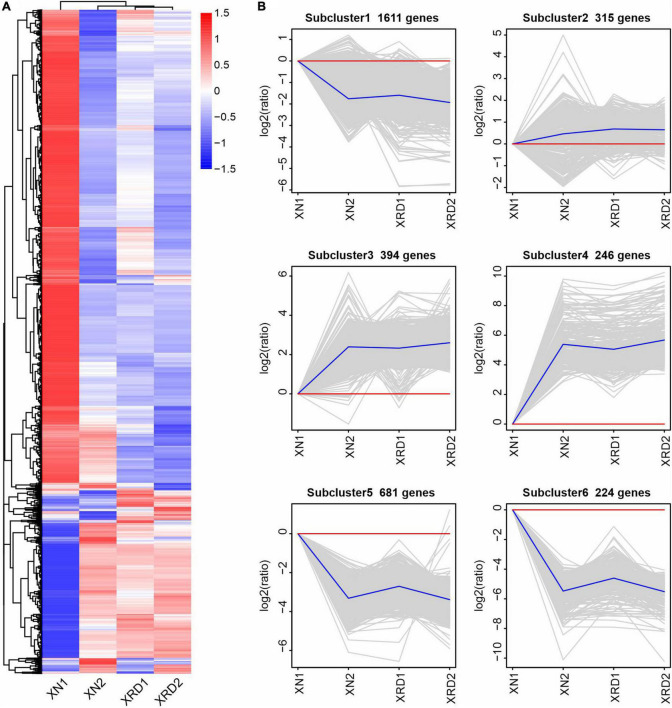
Differential gene H-cluster analysis and temporal dynamics analysis of the rapeseed transcriptome in XN and XRD at different time points. **(A)** Overall FPKM hierarchical cluster diagram normalized the log_10_(FPKM + 1) value and clusters it, red for high expression genes, and blue for low expression genes; The color is from red to blue, indicating that log_10_(FPKM + 1) is from large to small. **(B)** Log_2_(ratio) diagram of temporal dynamics analysis. The gray lines in each subgraph represent the relative expression of a gene in a cluster under different experimental conditions. The blue line represents the average relative expression of all genes in this cluster under different experimental conditions. The *x*-axis represents the experimental conditions, and the y-axis represents the relative expression.

To further investigate the transcriptomic dynamics at different time points in the XN and XRD areas, we also used the H-cluster (hierarchical clustering analysis) method to cluster the relative expression level of DEGs based on the FPKM values (Figure 4 and Supplementary Table 8). The results of the hierarchical clustering analysis showed that subcluster_1 (1,611 genes), subcluster_5 (681 genes), and subcluster_6 (224 genes) had the same trend of change, and the expression in both regions decreased after 40 days. The changing trend of subcluster_3 (394 genes) and subcluster_4 (246 genes) was the same, and the expression in both regions increased after 40 days. Subcluster_2 (315 genes) was relatively special, and the changes were relatively gentle, especially in the XRD area (Figure 3A). From the clustering results, XRD1, XRD2, and XN2 can be seen to have a stronger correlation (Figure 3B), indicating that the development of grains in the XRD region occurred significantly earlier and lasted longer than the development of grains in the XN region.

### Yield-related gene analysis

**FIGURE 5:**
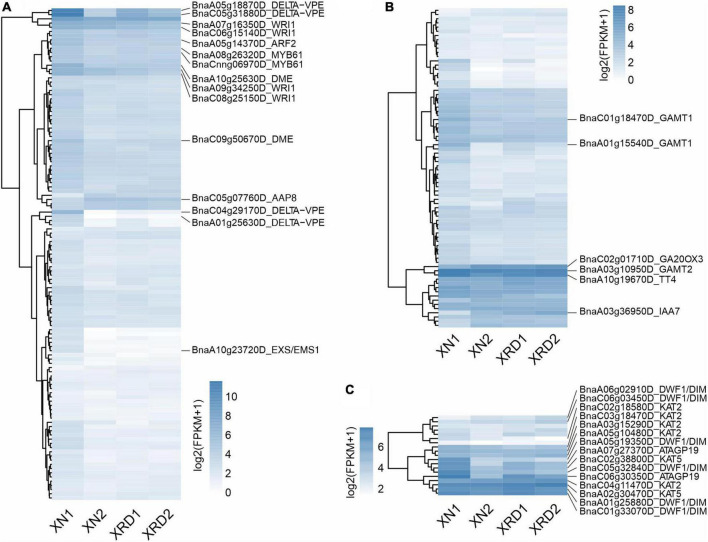
Expression profiles of genes implicated in yield traits. SW **(A)**, SY **(B)**, and PN **(C)**.

A previous study showed that a series of *Brassica napus* homologous genes of *Arabidopsis thaliana* yield-related genes were related to nine traits, such as SY (seed yield), SW (seed weight), and PN (pod number per plant) (Raboanatahiry et al., 2018). To further dissect the transcriptional profiles of genes involved in yield-related traits, we analyzed the expression profiles of genes related to SY (147), SW (189), and PN (28) during seed development in different areas (Figure 5 and Supplementary Table 9). As shown in Figure 5A, the main significant DEGs related to SW included delta vacuolar processing enzyme (DELTA-VPE, BnaC05g31880D, BnaA05g18870D, BnaA01g25630D, BnaC04g29170D), HhH-GPD base excision DNA repair family protein (DME, BnaA10g25630D, BnaC09g50670D), ethylene-responsive transcription factor (WRI1, BnaA07g16350D, BnaA09g34250D, BnaC08g25150D), and other DEGs, such as leucine-rich repeat transmembrane protein kinase (EXS/EMS1, BnaA10g23720D), amino acid permease 8 (AAP8, BnaC05g07760D), and MYB61 (BnaA08g26320D). The significant DEGs related to SY appeared mainly in hormone metabolism-related genes, such as the salicylic acid synthesis-degradation/C1-metabolism genes (BnaA01g15540D, BnaC01g18470D), alicyclic acid synthesis-degradation gene (BnaA03g10950D), gibberellin synthesis-degradation GA20 oxidase gene (BnaC02g01710D) and ethylene-related gene (RNA regulation of transcription Aux/IAA family, IAA7, BnaA03g36950D), and other significant DEGs, such as flavonoid chalcones naringenin-chalcone synthase gene (BnaA10g19670D) in secondary metabolism (Figure 5B). Among the PN-related genes shown in Figure 5C, there were three groups of significant DEGs, of which the first group contained delta (24)-sterol reductase genes (DWF1/DIM, BnaA05g19350D, BnaC05g32840D, BnaA06g02910D, BnaC01g33070D), the second group contained lysine-rich arabinogalactan protein (ATAGP19, BnaA07g27370D, BnaC06g30350D) and the third group includes 3-ketoacyl-CoA thiolase 5 (BnaC02g38800D, BnaA02g30470D) and 3-ketoacyl-CoA thiolase 2 (BnaC02g18580D, BnaC04g11470D).

Nine well-characterized genes, including *AUX1*, *CO*, *FT*, *FLC*, *PHYA*, *CRY2*, *AGL20*, *BRI1*, and *GAI*, were reported to be involved in key processes of crop yield determination, including the flowering process, light response, and plant hormone regulation (Geng et al., 2016, 2018; Lu et al., 2016, 2017; Pal et al., 2021). In this study, changes in these nine kinds of genes can be basically divided into three trends: the changes in the XN region are greater than the changes in the XRD region (standard changes); the changes in the XN region are less than the changes in the XRD region (special changes); and there is no change. Among them, PHYA (BnaC08g42660D) and CRY2 (BnaA10g02550D) are standard changes; AUX1 (BnaA05g06540D, auxin transport) and BRI1 (BnaA01g05490D, hormone metabolism, brassinosteroid signal transduction) also belong to standard changes, but GAI (BnaA02g12260D, hormone metabolism, gibberellin induced-regulated-responsive-activated) is unchanged. Among the other flowering-related genes, one FT (BnaA02g12130D) and AGL20 (BnaA03g56880D, RNA regulation of transcription MADS-box transcription factor family) belonged to standard changes, while CO (BnaA10g18430D) remained unchanged, but the remaining three genes, one FT (BnaC02g45250D) and two FLCs (BnaA02g00370D, BnaA03g02820D, RNA regulation of transcription MADS-box transcription factor family), belonged to special changes, which may play a special role in high yield in the XRD area (Supplementary Table 9).

**FIGURE 6:**
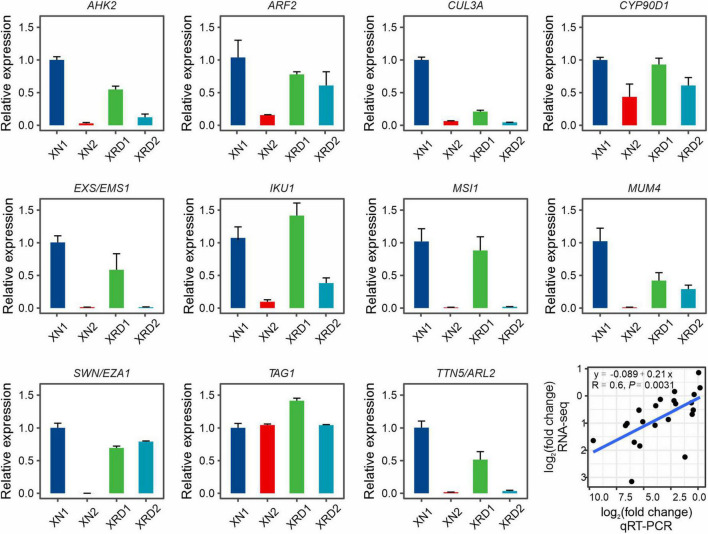
Expression profiles of yield-related genes in XN and XRD areas at 30 and 40 days based on qRT-PCR. Eleven yield-related genes with interests were selected. Correlation analysis showed that the qRT-PCR results were highly consistent with the RNA-seq results (*P* = 0.0031). Error bars indicate standard error (SE).

To validate the expression profiles of yield-related genes from RNA-Seq data, 11 yield-related genes, which were differentially expressed at different time points in XN and XRD, were selected for qRT-PCR analysis, each with three bioreplicates (Figure 6). The qRT-PCR primers were designed based on rapeseed sequences from BRAD data, and their primer sequences are listed in Supplementary Table 2. We compared the results obtained from qRT-PCR with the results generated from RNA-Seq analysis of these yield-related genes, resulting in a correlation coefficient of *R* = 0.6 and *P*-value = 0.0031 (Figure 6), indicating that expression trends were consistent for all transcripts in both analyses.

### Character seed filling stage by functional classification

**FIGURE 7:**
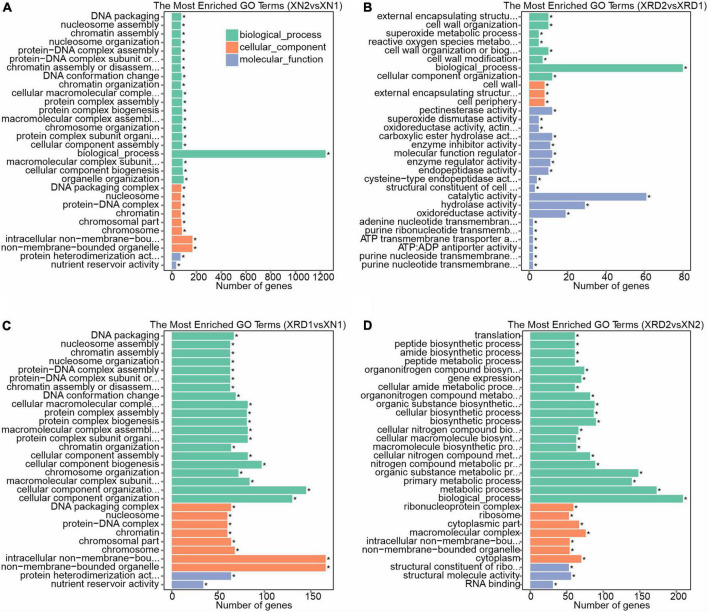
Gene function classification for DEGs identified in XN2 vs. XN1 **(A)**, XRD2 vs. XRD1 **(B)**, XRD1 vs. XN1 **(C)**, and XRD2 vs. XN2 **(D)**.

To characterize the putative functions and pathways of the DEGs of each sample, we carried out the GO enrichment analysis of DEGs using GOseq. To acquire complete functional information, GO terms were assigned to each DEG. Among all the identified DEGs between XN2 vs. XN1, XRD2 vs. XRD1, XRD1 vs. XN1, and XRD2 vs. XN2, 1,425, 409, 1,311, and 705 were annotated with GO terms, respectively (Figure 7 and Supplementary Table 10). Then, all DEGs were separated into upregulated and downregulated DEGs and functionally classified.

GO category analysis showed that DEGs identified in the XN area between the 30 vs. 40 DAF (XN2 vs. XN1) showed similar profiles for cellular component, biological process, and molecular function with XRD1 vs. XN1 (between XN and XRD area at the 30 DAF), with a high proportion of DEGs associated with nucleosome and chromosome in the cellular component. For the biological process category, DEGs were associated mainly with nucleosome assembly, chromatin assembly, and chromosome organization. For molecular function, protein heterodimerization activity and nutrient reservoir activity were the most highly represented categories (Figures 7A,C). In detail, among all the downregulated genes (1,349 out of 1,425 with GO annotations) between XN2 vs. XN1, 509, 167, 157, 130, 82, and 73 DEGs were assigned to heterocyclic compound binding (GO: 1901363), biosynthetic process (GO: 0009058), protein metabolic process (GO: 0019538), oxidation-reduction process (GO: 0055114), chromosome organization (GO: 0051276), and nucleosome assembly (GO: 0006334), respectively. In XRD1 vs. XN1, the downregulated genes (1,349 out of 1,425 with GO annotations) showed the same trend. In contrast, 464 upregulated genes between the 30 vs. 40 DAF in the XN area (XN2 vs. XN1) and 464 upregulated genes between the XN and XRD areas at the 30 DAF (XRD1 vs. XN1) showed different profiles. The 309, 80, 51, 72, 34, 22 up-DEGs related to biological process (GO: 0008150), oxidation-reduction process (GO: 0055114), small molecule metabolic process (GO: 0044281), oxidoreductase activity (GO: 0016491), nutrient reservoir activity (nutrient reservoir activity), lipid particle (GO: 0005811) in XN2 vs. XN1, while 296, 88, 75, 66, 47, 46, 33, 32, 27, 27, 22,19 up-DEGs related to biological process (GO: 0008150), membrane (GO: 0016020), oxidation-reduction process (GO: 0055114), oxidoreductase activity (GO: 0016491), carbohydrate metabolic process (GO: 0005975), small molecule metabolic process (GO: 0044281), nutrient reservoir activity (GO: 0045735), lipid metabolic process (GO: 0006629), proteolysis (GO: 0006508), response to stimulus (GO: 0050896), lipid particle (GO: 0005811), cell wall organization or biogenesis (GO: 0071554) in XRD1 vs. XN1.

Most of the DEGs between the same XRD area (XRD2 vs. XRD1) were assigned to biological process, catalytic activity, hydrolase activity, and oxidoreductase activity, followed by cell wall, cell wall organization or biogenesis, reactive oxygen species (ROS) metabolic process, pectinesterase activity, carboxylic ester hydrolase activity, enzyme inhibitor activity, molecular function regulator, enzyme regulator activity, and endopeptidase activity (Figure 7B). In detail, among all the downregulated genes (50 out of 103 with GO annotations) in XRD2 vs XRD1, 18, 8, 8, 8, 6, 4, 4, and 4 DEGs were assigned to hydrolase activity (GO: 0016787), pectinesterase activity (GO: 0030599), carboxylic ester hydrolase activity (GO: 0052689), endopeptidase activity (GO: 0004175), enzyme inhibitor activity (GO: 0004857), photosystem (GO: 0009521), thylakoid (GO: 0009579), and photosynthesis (GO: 0015979), respectively. In contrast, 46 out of 53 upregulated genes in XRD2 vs. XRD1 were assigned to biological process (GO: 0008150), 14, 9, and 6 upregulated DEGs related to oxidoreductase activity (GO: 0016491), cellular component organization (GO: 0016043), and molecular function regulator (GO: 0098772), and 7, 4 upregulated DEGs related to cell wall organization (GO: 0071555) and cell wall (GO: 0005618), respectively.

Most of the DEGs between the same 40 DAF stage of XN and XRD seeds (XRD2 vs. XN2) were also assigned to biological process, metabolic process, macromolecular complex, cytoplasm, structural molecule activity followed by gene expression, peptide biosynthetic process, translation, ribosome, structural constituent of ribosome, and RNA binding (Figure 7D). In detail, among all the downregulated genes (179 out of 280 with GO annotations) in XRD2 vs XN2, 103, 78, 70, 61, 54, and 53 DEGs were assigned to the primary metabolic process (GO: 0044238), cellular biosynthetic process (GO: 0044249), gene expression (GO: 0010467), translation (GO: 0006412), structural molecule activity (GO: 0005198), and ribosome (GO: 0005840), respectively. In contrast, 64, 29, 22, 16, and 7 upregulated genes in XRD2 vs. XN2 were assigned to catalytic activity (GO: 0003824), hydrolase activity (GO: 0016787), membrane (GO: 0016020), proteolysis (GO: 0006508), and cell wall organization (GO: 0071555).

**FIGURE 8:**
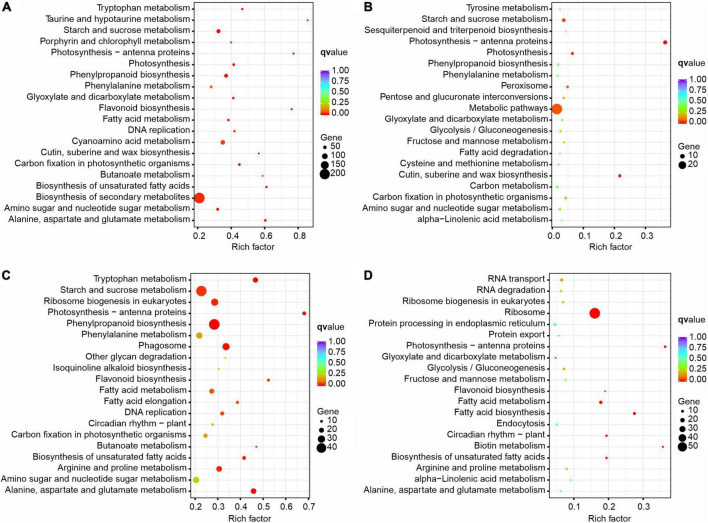
KEGG enrichment of DEGs identified from pairwise comparisons in XN2 vs. XN1 **(A)**, XRD2 vs. XRD1 **(B)**, XRD1 vs. XN1 **(C)**, and XRD2 vs. XN2 **(D)**.

A pathway-based analysis will help us to further understand the biological functions of DEGs. To identify metabolic pathways in which DEGs were involved and enriched, the KEGG was used to analyze all the DEGs by identifying the top 20 most enriched KEGG pathways with KOBAS 2.0 (Figure 8 and Supplementary Table 11). In the comparison of different times in the XN area (XN2 vs XN1), biosynthesis of secondary metabolites was the main enrichment pathway, followed by starch and sucrose metabolism, phenylpropanoid biosynthesis, amino sugar and nucleotide sugar metabolism, and photosynthesis. In detail, metabolic pathways, starch and sucrose metabolism, amino sugar and nucleotide sugar metabolism, and photosynthesis were enriched mainly with downregulated DEGs in XN2 vs. XN1. In contrast, the upregulated DEGs in XN2 vs. XN1 were most significantly enriched in the biosynthesis of secondary metabolites, followed by phenylpropanoid biosynthesis and fatty acid metabolism (Figure 8A).

In the comparison of different times in the XRD area (XRD2 vs. XRD1), metabolic pathways were the main enrichment pathway, followed by photosynthesis-antenna proteins, cutin, suberin, and wax biosynthesis, photosynthesis, starch, and sucrose metabolism. In detail, metabolic pathways, photosynthesis-antenna proteins, photosynthesis, starch, and sucrose metabolism were mainly enriched with downregulated DEGs in XRD2 vs XRD1. In contrast, the upregulated DEGs in XRD2 vs. XRD1 were most significantly enriched in metabolic pathways, followed by biosynthesis of secondary metabolites, cutin, suberine and wax biosynthesis, and peroxisome (Figure 8B).

In comparing different areas at 30DAF (XRD1 vs. XN1), starch and sucrose metabolism was the main enrichment pathway, followed by phenylpropanoid biosynthesis, ribosome biogenesis in eukaryotes, phagosome, arginine and proline metabolism, fatty acid metabolism. In detail, starch and sucrose metabolism, ribosome biogenesis in eukaryotes, phagosome, amino sugar and nucleotide sugar metabolism, and arginine and proline metabolism were enriched mainly with downregulated DEGs in XRD1 vs. XN1. In contrast, the upregulated DEGs in XRD1 vs. XN1 were most significantly enriched in the biosynthesis of secondary metabolites, followed by phenylpropanoid biosynthesis and tryptophan metabolism (Figure 8C).

In the comparison of different areas at 40DAF (XRD2 vs. XN2), the ribosome was the main enrichment pathway, followed by fatty acid metabolism, photosynthesis-antenna proteins, and circadian rhythm – plant. In detail, ribosome, fatty acid metabolism, biotin metabolism, circadian rhythm-plant, and RNA transport were noted to be more highly enriched with downregulated DEGs in XN2 vs. XN1. In contrast, the upregulated DEGs in XN2 vs. XN1 were most significantly enriched in metabolic pathways, followed by photosynthesis-antenna proteins, flavonoid biosynthesis, and alanine, aspartate, and glutamate metabolism (Figure 8D).

### Rapid initiation into late seed development triggered by long sunshine is the key to high yield

**FIGURE 9:**
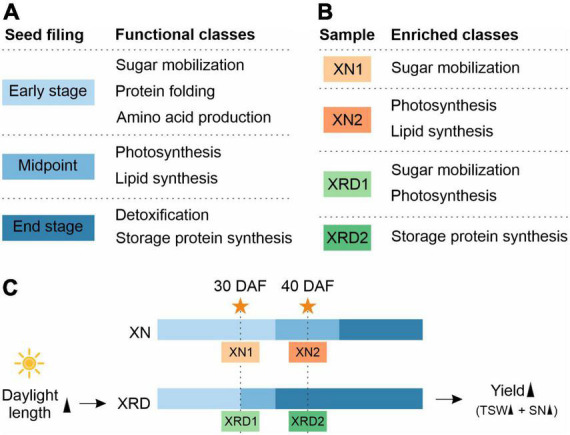
A proposed working model for rapeseed high yield in the unique plateau habitat.

A previous study has suggested that cellular activity during seed filling in *B. napus* begins with sugar mobilization, followed by sequential surges in amino acid, lipid, and storage protein synthesis (Hajduch et al., 2006). They divided the expression trends into different functional subclasses based on three seed filling stages (Figure 9A). The first group included proteins expressed mainly at the early stages of seed filling, including proteins involved in glycolysis, respiration, metabolism of sugars, signal transduction, metabolism of amino acids, proteolysis, and defense. Proteins of the second group, involved in photosynthesis and lipid metabolism, exhibited the highest expression at the midpoint of seed filling. Finally, detoxification, seed maturation, and seed storage proteins were highly abundant at the end stage of seed filling.

Combining the above results from GO and KEGG pathway analyses and H-cluster analysis of DEGs, we can speculate that the seed developmental period in each sample is as follows (Figure 9B and 9C): 1) XN1 belongs to the early stage of expression, responsible mainly for starch and sucrose metabolism, nucleosome assembly, and chromatin assembly; 2) XN2 belongs to the midpoint stage of expression, accountable mainly for ribosome, fatty acid metabolism, photosynthesis, gene expression, and translation; 3) XRD1 belongs to the intermediate stage between the first and second stages of expression, responsible mainly for photosynthesis, starch and sucrose metabolism, cell wall organization or biogenesis, reactive oxygen species metabolic process, and enzyme regulator activity; and 4) XRD2 belongs to the end stage of seed filling, in which DEGs were enriched in storage protein synthesis. Taken together, our data suggest that seed development in the XRD region occurred significantly earlier and lasted longer, which was caused by a relatively longer daylight length, resulting in a higher yield in the Xiangride area.

## Discussion

Although numerous studies have focused on seed yield genetic improvement of rapeseed through quantitative trait locus (QTL) mapping, high-throughput sequencing, and genotyping techniques (Yang et al., 2012; Liu et al., 2015b; Zhao et al., 2016; Shah et al., 2018; Zheng et al., 2020; Khan et al., 2021; Hu et al., 2022), the comprehensive study of seed development and the mechanism contributing to high yield in the high-yield field, especially the Xiangride area, Tsaldam Basin of Qinghai Province in China, is poor, which is a constraint to our in-depth understanding of the environmental influences of this important trait and provides valuable information for future rapeseed breeding.

### Relationship between high rapeseed yield and the unique eco-environment

Since Maskell and Mason proposed the source-sink theory to describe yield formation through the study of carbohydrate distribution in cotton plants in 1928, the methods of high yield from the source-sink point of view have often been discussed (Diepenbrock, 2000; Slafer, 2003). Subsequently, some crop traits were introduced into high yield formation; that is, crop yield is directly and multiply determined by yield component traits, including silique length (SL), seed weight/thousand seed weight (SW/TSW), SPS, and siliques per plant (SP), which are closely associated with seed yield improvement. Yield-related traits (such as biomass, harvest index, plant architecture, adaptation, and resistance to biotic and abiotic constraints) may also indirectly affect yield by affecting yield-component traits or by other unknown mechanisms (Diepenbrock, 2000; Chen et al., 2007). With the variety of crops, the yield components of high-yielding performance are different. In spring rapeseed, the high yield mainly showed that the number of pods per plant and 1,000-grain weight increased significantly, but the overall response was the synergistic effect of “source-intensive” and “sink-intensive” (Diepenbrock, 2000; Shi et al., 2009; Luo et al., 2015; Lu et al., 2016). In this study, we found that the SN and TSW in the XRD area were significantly greater than the SN and TSW in the XN area (Figure 1). This result showed that these two traits were the main factors resulting in high yield in the specific eco-environment of the XRD reproducing area.

The formation and potential development of high crop yields are greatly influenced by the distribution of photosynthetic assimilation among different organs and tissues. In the process of seed development, grain filling is an important stage to determine grain yield and is also a crucial and final stage of plant growth. Seed-filling duration is the morphology and physiological metabolism of rapeseed that will change dramatically in the process of grain filling, involving the supply of various components and precursors from the leaves into developing seeds, and diverse biochemical processes take place to synthesize carbohydrates, proteins, and lipids (Triboi et al., 2003; Barnabas et al., 2008; Awasthi et al., 2014). The yield and quality of grains are determined by the amount of organic matter synthesized or stored and the direction of transport, especially the photosynthate transport from the pod to seeds (Diepenbrock, 2000; Hajduch et al., 2006; Liu et al., 2015a). The production and transportation of photosynthetic assimilation are affected not only by the characteristics of the crops themselves but also by biotic or abiotic factors in the environment. Yield reflects the interaction of the environment with all growth and development processes that occur throughout the life cycle (Quarrie et al., 2006). In other words, yield is related to the environment; that is, seed development and even grain filling related to yield are also related to the environment. Many environmental conditions affect seed development, such as temperature, rainfall, light duration, and soil conditions (Barnabas et al., 2008; Huner et al., 2014; Fletcher et al., 2015; Macova et al., 2022). Some research results indicate that the high potential yield of irrigated rice in Yunnan is achieved mainly by intense incident solar radiation, which causes large biomass and N accumulation. The low nighttime temperature during the midgrowth stage was also suggested to be an important factor for considerable biomass accumulation and high grain yield in Yunnan (Ying et al., 1998a,b; Katsura et al., 2008). A high crop growth rate results from greater apparent canopy photosynthesis during daylight and less maintenance respiration at night (Sadras et al., 1993). Many experiments that compare the growth of a genotype in current and future projected elevated [CO_2_] environments show that an increase in leaf photosynthesis is closely associated with similar increases in yield (Long et al., 2006). The physiological characteristics of high-yield crops in special ecological environment are reflected mainly in the improvement of net assimilation capacity, the extension of the functional period of photosynthetic organs after anthesis, the delay of photosynthetic organ senescence, and the large storage capacity, (Ying et al., 1998a,b; Long et al., 2006). In this study, the main advantage of the XRD area compared with the XN area is found to be that the sunshine length was longer, while other environmental factors (average temperature, rainfall, and average soil temperature of 20 cm) were lower than the environmental factors in the Xining area. Therefore, we suggest that the high yield of special habitats may be due to the extension of sunshine time, and we can hypothesize that the specific eco-environment of the XRD area would lead to an increase in day length, which leads to the rise in the yield per plant.

### Changes in yield candidate genes and metabolic pathways during seed development in *Brassica napus* L

Seed yield is a complex trait and can be improved by the direct component traits and the other indirect contributing traits. Both direct yield component traits and indirect yield-related traits are elaborately controlled by several genes and are highly influenced by environmental conditions (Van Camp, 2005; Shi et al., 2009; Ding et al., 2012; Zhao et al., 2016; Lu et al., 2017). The identification of yield candidate genes implies the detection of important genes for agricultural and economic quantitative traits (Raboanatahiry et al., 2018, 2022; Hu et al., 2021, 2022; Pal et al., 2021; Zheng et al., 2022). In the model plant *Arabidopsis thaliana*, 425 genes were collected from the TAIR website^2^ to be related to flowering time, plant height, branch number, seed number, seed weight, and SY (Shi et al., 2009). In *B. napus*, several studies on yield candidate genes have previously been performed; for instance, Zhao et al. (2016) found four and two candidate genes for SY and SW traits (Shi et al., 2009; Zhao et al., 2016). Additionally, candidate genes for PN per plant (seven genes), branch PN (seven genes), and 1000 seed weights (one gene) were discovered (Lu et al., 2017). Moreover, a total of 1,562 genes in *B. napus* were homologous of 425 yield-related genes in *Arabidopsis thaliana*, and a total of 147 candidate genes were found inside regions of overlapping QTLs for nine traits, including PN, SW, and SY. Then, 147 SY-related, 189 SW-related, and 28 PN-related *Brassica napus* homologous genes of *Arabidopsis thaliana* yield-related genes were found (Raboanatahiry et al., 2018). Based on the construction of a quantitative genomic map of *Brassica napus*, Raboanatahiry further revealed 517 regions of overlapping QTLs that harbored 2,744 candidate genes and might affect multiple traits simultaneously (Raboanatahiry et al., 2022). Hu unraveled the genomic basis for the selection of adaptation and agronomic traits and identified 628 associated locus-related causative candidate genes for 56 agronomically important traits (including plant architecture and yield traits) through genome-wide association studies (Hu et al., 2022). In this study, we used these rapeseed candidate yield-related genes in our transcriptome data analysis and the results showed that the DEGs related to yield traits were generally scattered changes, which means that the high-yield environment may affect rapeseed yield indirectly.

Recent technical advancements in transcriptomics have provided new ways of addressing the temporal and spatial changes in gene expression associated with seed maturation and yield-determining traits (Harper et al., 2012; Liu et al., 2015a; Lu et al., 2016; Geng et al., 2018; Canales et al., 2021). Here, we carried out a transcriptome analysis of rapeseed seeds under XN and XRD high-altitude environmental conditions. Based on previous studies, the gene expression trends during seed filling in *B. napus* can be divided into three different functional stages, beginning with sugar mobilization, followed by sequential surges in photosynthesis and lipid metabolism, and finally, detoxification, seed maturation, and storage protein synthesis (Hajduch et al., 2006; Huang et al., 2013). In this study, compared with the filling development stage of rapeseeds, combined with transcriptome H-cluster analysis and GO and KEGG functional analyses, we found that the third development stage of rapeseeds in the XRD area, namely, the storage protein synthesis stage, was significantly ahead of schedule and lasted for a long time, resulting in a high XRD yield. Overall, based on the comparative analysis of environmental conditions and yields, we can infer that changes in environmental conditions, such as an increase in altitude, lead to an increase in sunshine length, and an increase in sunshine length leads to changes in the stage of rapeseed filling development and ultimately leads to an increase in rapeseed yield.

## Conclusion

We conducted field yield analysis and transcriptome analysis in Xiangride, a special high-yield ecological area of rapeseed on the Qinghai Plateau. Compared with the yield and environmental factors in the Xining area, we found that the extension of daylight time in the Xiangride area may lead to an increase in rapeseed yield and significantly increases in seed weight (SW) and SN. Combined with transcriptome H-cluster analysis and GO and KEGG functional analyses, we can assume that the grain development of rapeseed in the Xiangride area is ahead of schedule and lasts for a long time, leading to the high-yield results in the Xiangride area, confirmed by the expression analysis by qRT-PCR of yield-related genes. These results suggest that the unique pattern of seed development in the XRD region, which developed ahead in the filling stage, was caused by a relatively longer daylight length, which is the key to high rapeseed yield in the Xiangride area. Our results provide valuable information for further exploring the molecular mechanism of high yield in special ecological environments and provide a useful reference for studying seed development characteristics in special regions of *B. napus*. At the same time, this study also provides a theoretical basis for how to improve high crop yield by adjusting the agricultural environment of rapeseed in the future.

## Data availability statement

The datasets presented in this study can be found in online repositories. The names of the repository/repositories and accession number(s) can be found below: Sequenced reads are available 573 in NCBI SRA (sequence read archive) under accession SRR 18827069 to SRR 18827072, 574 (https://www.ncbi.nlm.nih.gov/bioproject/PRJNA828284).

## Author contributions

HX and RD designed the study. HX, HS, and RW performed experiments. HX, RD, and XJ analyzed the RNA-seq data and drafted the manuscript. HX, XJ, and RD revised the manuscript. All authors contributed to the article and approved the submitted version.

## Footnotes

1
http://www.cma.gov.cn/

2
http://www.arabidopsis.org/

## References

[B1] Awasthi R. Kaushal N. Vadez V. Turner N. C. Berger J. Siddique K. H. M. (2014). Individual and combined effects of transient drought and heat stress on carbon assimilation and seed filling in chickpea. *Funct. Plant Biol.* 41 1148–1167. 10.1071/FP13340 32481065

[B2] Barnabas B. Jager K. Feher A. (2008). The effect of drought and heat stress on reproductive processes in cereals. *Plant Cell Environ.* 31 11–38. 17971069 10.1111/j.1365-3040.2007.01727.x

[B3] Basnet R. K. Moreno-Pachon N. Lin K. Bucher J. Visser R. G. Maliepaard C. (2013). Genome-wide analysis of coordinated transcript abundance during seed development in different *Brassica rapa* morphotypes. *BMC Genomics* 14:840. 10.1186/1471-2164-14-840 24289287 PMC4046715

[B4] Borisjuk L. Neuberger T. Schwender J. Heinzel N. Sunderhaus S. Fuchs J. (2013). Seed architecture shapes embryo metabolism in oilseed rape. *Plant Cell* 25 1625–1640. 10.1105/tpc.113.111740 23709628 PMC3694696

[B5] Canales J. Verdejo J. Carrasco-Puga G. Castillo F. M. Arenas M. A. Calderini D. F. (2021). Transcriptome analysis of seed weight plasticity in *Brassica napus*. *Int. J. Mol. Sci.* 22:4449. 10.3390/ijms22094449 33923211 PMC8123204

[B6] Chalhoub B. Denoeud F. Liu S. Parkin I. A. Tang H. Wang X. (2014). Plant genetics. Early allopolyploid evolution in the post-Neolithic *Brassica napus* oilseed genome. *Science* 345 950–953. 10.1126/science.1253435 25146293

[B7] Chen W. Zhang Y. Liu X. Chen B. Tu J. Tingdong F. (2007). Detection of QTL for six yield-related traits in oilseed rape (*Brassica napus*) using DH and immortalized F(2) populations. *Theor. Appl. Genet.* 115 849–858. 10.1007/s00122-007-0613-2 17665168

[B8] Diepenbrock W. (2000). Yield analysis of winter oilseed rape (*Brassica napus* L.): a review. *Field Crops Res.* 67 35–49.

[B9] Ding G. Zhao Z. Liao Y. Hu Y. Shi L. Long Y. (2012). Quantitative trait loci for seed yield and yield-related traits, and their responses to reduced phosphorus supply in *Brassica napus*. *Ann. Bot.* 109 747–759. 10.1093/aob/mcr323 22234558 PMC3286287

[B10] Fletcher R. S. Mullen J. L. Heiliger A. Mckay J. K. (2015). QTL analysis of root morphology, flowering time, and yield reveals trade-offs in response to drought in *Brassica napus*. *J. Exp. Bot.* 66 245–256. 10.1093/jxb/eru423 25371500 PMC4265167

[B11] Fu Y. Wei D. Dong H. He Y. Cui Y. Mei J. (2015). Comparative quantitative trait loci for silique length and seed weight in *Brassica napus*. *Sci. Rep.* 5:14407. 10.1038/srep14407 26394547 PMC4585775

[B12] Geng X. Dong N. Wang Y. Li G. Wang L. Guo X. (2018). RNA-seq transcriptome analysis of the immature seeds of two *Brassica napus* lines with extremely different thousand-seed weight to identify the candidate genes related to seed weight. *PLoS One* 13:e0191297. 10.1371/journal.pone.0191297 29381708 PMC5790231

[B13] Geng X. Jiang C. Yang J. Wang L. Wu X. Wei W. (2016). Rapid identification of candidate genes for seed weight using the SLAF-Seq method in *Brassica napus*. *PLoS One* 11:e0147580. 10.1371/journal.pone.0147580 26824525 PMC4732658

[B14] Gupta M. Bhaskar P. B. Sriram S. Wang P. H. (2017). Integration of omics approaches to understand oil/protein content during seed development in oilseed crops. *Plant Cell Rep.* 36 637–652. 10.1007/s00299-016-2064-1 27796489

[B15] Hajduch M. Casteel J. E. Hurrelmeyer K. E. Song Z. Agrawal G. K. Thelen J. J. (2006). Proteomic analysis of seed filling in Brassica napus. Developmental characterization of metabolic isozymes using high-resolution two-dimensional gel electrophoresis. *Plant Physiol.* 141 32–46. 10.1104/pp.105.075390 16543413 PMC1459325

[B16] Harper A. L. Trick M. Higgins J. Fraser F. Clissold L. Wells R. (2012). Associative transcriptomics of traits in the polyploid crop species *Brassica napus*. *Nat. Biotechnol.* 30 798–802. 10.1038/nbt.2302 22820317

[B17] Hay J. Schwender J. (2011). Metabolic network reconstruction and flux variability analysis of storage synthesis in developing oilseed rape (*Brassica napus* L.) embryos. *Plant J.* 67 526–541. 10.1111/j.1365-313X.2011.04613.x 21501263

[B18] Hu D. Jing J. Snowdon R. J. Mason A. S. Shen J. Meng J. (2021). Exploring the gene pool of *Brassica napus* by genomics-based approaches. *Plant Biotechnol. J.* 19 1693–1712. 10.1111/pbi.13636 34031989 PMC8428838

[B19] Hu J. Chen B. Zhao J. Zhang F. Xie T. Xu K. (2022). Genomic selection and genetic architecture of agronomic traits during modern rapeseed breeding. *Nat. Genet.* 54 694–704. 10.1038/s41588-022-01055-6 35484301

[B20] Hu Y. Wu G. Cao Y. Wu Y. Xiao L. Li X. (2009). Breeding response of transcript profiling in developing seeds of *Brassica napus*. *BMC Mol. Biol.* 10:49. 10.1186/1471-2199-10-49 19463193 PMC2697984

[B21] Huang D. Koh C. Feurtado J. A. Tsang E. W. Cutler A. J. (2013). MicroRNAs and their putative targets in *Brassica napus* seed maturation. *BMC Genomics* 14:140. 10.1186/1471-2164-14-140 23448243 PMC3602245

[B22] Huner N. P. Dahal K. Kurepin L. V. Savitch L. Singh J. Ivanov A. G. (2014). Potential for increased photosynthetic performance and crop productivity in response to climate change: role of CBFs and gibberellic acid. *Front. Chem.* 2:18. 10.3389/fchem.2014.00018 24860799 PMC4029004

[B23] Katsura K. Maeda S. Lubis I. Horie T. Cao W. Shiraiwa T. (2008). The high yield of irrigated rice in Yunnan, China: ‘A cross-location analysis’. *Field Crops Res.* 107 1–11.

[B24] Khan S. U. Saeed S. Khan M. H. U. Fan C. Ahmar S. Arriagada O. (2021). Advances and challenges for QTL analysis and GWAS in the plant-breeding of high-yielding: a focus on rapeseed. *Biomolecules* 11:1516. 10.3390/biom11101516 34680149 PMC8533950

[B25] Leon J. (1993). The importance of crop physiology for the breeding of oilseed rape. *Fett* 95 283–287.

[B26] Li N. Shi J. Wang X. Liu G. Wang H. (2014). A combined linkage and regional association mapping validation and fine mapping of two major pleiotropic QTLs for seed weight and silique length in rapeseed (*Brassica napus* L.). *BMC Plant Biol.* 14:114. 10.1186/1471-2229-14-114 24779415 PMC4021082

[B27] Liu H. Yang Q. Fan C. Zhao X. Wang X. Zhou Y. (2015a). Transcriptomic basis of functional difference and coordination between seeds and the silique wall of *Brassica napus* during the seed-filling stage. *Plant Sci.* 233 186–199. 10.1016/j.plantsci.2015.01.015 25711826

[B28] Liu J. Hua W. Hu Z. Yang H. Zhang L. Li R. (2015b). Natural variation in ARF18 gene simultaneously affects seed weight and silique length in polyploid rapeseed. *Proc. Natl. Acad. Sci. U.S.A.* 112 E5123. 10.1073/pnas.1502160112 26324896 PMC4577148

[B29] Long S. P. Zhu X. G. Naidu S. L. Ort D. R. (2006). Can improvement in photosynthesis increase crop yields? *Plant Cell Environ.* 29 315–330. 17080588 10.1111/j.1365-3040.2005.01493.x

[B30] Lu K. Peng L. Zhang C. Lu J. Yang B. Xiao Z. (2017). Genome-wide association and transcriptome analyses reveal candidate genes underlying yield-determining traits in *Brassica napus*. *Front. Plant Sci.* 8:206. 10.3389/fpls.2017.00206 28261256 PMC5309214

[B31] Lu K. Xiao Z. Jian H. Peng L. Qu C. Fu M. (2016). A combination of genome-wide association and transcriptome analysis reveals candidate genes controlling harvest index-related traits in *Brassica napus*. *Sci. Rep.* 6:36452. 10.1038/srep36452 27811979 PMC5095561

[B32] Luo X. Ma C. Yue Y. Hu K. Li Y. Duan Z. (2015). Unravelling the complex trait of harvest index in rapeseed (*Brassica napus* L.) with association mapping. *BMC Genomics* 16:379. 10.1186/s12864-015-1607-0 25962630 PMC4427920

[B33] Luo Z. Wang M. Long Y. Huang Y. Shi L. Zhang C. (2017). Incorporating pleiotropic quantitative trait loci in dissection of complex traits: seed yield in rapeseed as an example. *Theor. Appl. Genet.* 130 1569–1585. 28455767 10.1007/s00122-017-2911-7 PMC5719798

[B34] Macova K. Prabhullachandran U. Stefkova M. Spyroglou I. Pencik A. Endlova L. (2022). Long-term high-temperature stress impacts on embryo and seed development in *Brassica napus*. *Front. Plant Sci.* 13:844292. 10.3389/fpls.2022.844292 35528932 PMC9075611

[B35] Mason A. S. Snowdon R. J. (2016). Oilseed rape: learning about ancient and recent polyploid evolution from a recent crop species. *Plant Biol.* 18 883–892. 10.1111/plb.12462 27063780

[B36] Pal L. Sandhu S. K. Bhatia D. Sethi S. (2021). Genome-wide association study for candidate genes controlling seed yield and its components in rapeseed (*Brassica napus* subsp. napus). *Physiol. Mol. Biol. Plants* 27 1933–1951. 10.1007/s12298-021-01060-9 34629771 PMC8484396

[B37] Quarrie S. Pekic Quarrie S. Radosevic R. Rancic D. Kaminska A. Barnes J. D. (2006). Dissecting a wheat QTL for yield present in a range of environments: from the QTL to candidate genes. *J. Exp. Bot.* 57 2627–2637. 10.1093/jxb/erl026 16831847

[B38] Quijada P. A. Udall J. A. Lambert B. Osborn T. C. (2006). Quantitative trait analysis of seed yield and other complex traits in hybrid spring rapeseed (*Brassica napus* L.): 1. Identification of genomic regions from winter germplasm. *Theor. Appl. Genet.* 113 549–561. 10.1007/s00122-006-0323-1 16767447

[B39] Raboanatahiry N. Chao H. Dalin H. Pu S. Yan W. Yu L. (2018). QTL alignment for seed yield and yield related traits in *Brassica napus*. *Front. Plant Sci.* 9:1127. 10.3389/fpls.2018.01127 30116254 PMC6083399

[B40] Raboanatahiry N. Chao H. He J. Li H. Yin Y. Li M. (2022). Construction of a quantitative genomic map, identification and expression analysis of candidate genes for agronomic and disease-related traits in *Brassica napus*. *Front. Plant Sci.* 13:862363. 10.3389/fpls.2022.862363 35360294 PMC8963808

[B41] Sadras V. O. Hall A. J. Connor D. J. (1993). Light-associated nitrogen distribution profile in flowering canopies of sunflower (*Helianthus annuus* L.) altered during grain growth. *Oecologia* 95 488–494. 10.1007/BF00317432 28313288

[B42] Shah S. Karunarathna N. L. Jung C. Emrani N. (2018). An APETALA1 ortholog affects plant architecture and seed yield component in oilseed rape (*Brassica napus* L.). *BMC Plant Biol.* 18:380. 10.1186/s12870-018-1606-9 30594150 PMC6310979

[B43] Shi J. Li R. Qiu D. Jiang C. Long Y. Morgan C. (2009). Unraveling the complex trait of crop yield with quantitative trait loci mapping in *Brassica napus*. *Genetics* 182 851–861. 10.1534/genetics.109.101642 19414564 PMC2710164

[B44] Shi S. B. Chen W. J. Shi R. Li M. Zhang H. G. Sun Y. N. (2014). PS II photochemical efficiency in flag leaf of wheat varieties and its adaptation to strong sun- light intensity on farmland of Xiangride in Qinghai Province, Northwest China. *Ying Yong Sheng Tai Xue Bao* 25 2613–2622. 25757313

[B45] Slafer G. A. (2003). Genetic basis of yield as viewed from a crop physiologist’s perspective. *Ann. Appl. Biol.* 142 117–128.

[B46] Su T. Pan Z. (1981). An analysis of the physiological features of the higher yielding ability of spring wheat in the Xiangride farm, Qinghai province. *Acta Agron. Sin.* 1 19–26.

[B47] Triboi E. Martre P. Triboi-Blondel A. M. (2003). Environmentally-induced changes in protein composition in developing grains of wheat are related to changes in total protein content. *J. Exp. Bot.* 54 1731–1742. 10.1093/jxb/erg183 12773520

[B48] Udall J. A. Quijada P. A. Lambert B. Osborn T. C. (2006). Quantitative trait analysis of seed yield and other complex traits in hybrid spring rapeseed (*Brassica napus* L.): 2. Identification of alleles from unadapted germplasm. *Theor. Appl. Genet.* 113 597–609. 10.1007/s00122-006-0324-0 16767446

[B49] Van Camp W. (2005). Yield enhancement genes: seeds for growth. *Curr. Opin. Biotechnol.* 16 147–153. 15831379 10.1016/j.copbio.2005.03.002

[B50] Yang P. Shu C. Chen L. Xu J. Wu J. Liu K. (2012). Identification of a major QTL for silique length and seed weight in oilseed rape (*Brassica napus* L.). *Theor. Appl. Genet.* 125 285–296. 10.1007/s00122-012-1833-7 22406980

[B51] Ying J. Peng S. He Q. Yang H. Yang C. Visperas R. M. (1998a). Comparison of high-yield rice in tropical and subtropical environments: I. Determinants of grain and dry matter yields. *Field Crops Res.* 57 71–84.

[B52] Ying J. Peng S. Yang G. Zhou N. Visperas R. M. Cassman K. G. (1998b). Comparison of high-yield rice in tropical and subtropical environments: II. Nitrogen accumulation and utilization efficiency. *Field Crops Res.* 57 85–93.

[B53] Zhao W. Wang X. Wang H. Tian J. Li B. Chen L. (2016). Genome-wide identification of QTL for seed yield and yield-related traits and construction of a high-density consensus map for QTL comparison in *Brassica napus*. *Front. Plant Sci.* 7:17. 10.3389/fpls.2016.00017 26858737 PMC4729939

[B54] Zhao X. Yu K. Pang C. Wu X. Shi R. Sun C. (2021). QTL analysis of five silique-related traits in *Brassica napus* L. Across multiple environments. *Front. Plant Sci.* 12:766271. 10.3389/fpls.2021.766271 34887891 PMC8650614

[B55] Zheng M. Peng C. Liu H. Tang M. Yang H. Li X. (2017). Genome-wide association study reveals candidate genes for control of plant height, branch initiation height and branch number in rapeseed (*Brassica napus* L.). *Front. Plant Sci.* 8:1246. 10.3389/fpls.2017.01246 28769955 PMC5513965

[B56] Zheng M. Terzaghi W. Wang H. Hua W. (2022). Integrated strategies for increasing rapeseed yield. *Trends Plant Sci.* S1360–1385, 00080–00082. 10.1016/j.tplants.2022.03.008 35501261

[B57] Zheng M. Zhang L. Tang M. Liu J. Liu H. Yang H. (2020). Knockout of two BnaMAX1 homologs by CRISPR/Cas9-targeted mutagenesis improves plant architecture and increases yield in rapeseed (*Brassica napus* L.). *Plant Biotechnol. J.* 18 644–654. 10.1111/pbi.13228 31373135 PMC7004912

