# Supplementary material for: Transcriptomic analysis of rapeseed (Brassica napus. L.) seed development in Xiangride, Qinghai Plateau, reveals how its special eco-environment results in high yield in high-altitude areas
Source: Front Plant Sci. 2022 Aug 2;13:927418. doi: 10.3389/fpls.2022.927418 (PMC9379305; doi:10.3389/fpls.2022.927418)
Supplement: Supplementary file 1 [file Data_Sheet_1.docx]

**Supplementary Material**

Additional file 1: Table S1. Climate data of XN and XRD producing areas in 2015.

Additional file 2: Table S2. The primer sequences.

Additional file 3: Table S3. Changes in yield-related traits.

Additional file 4: Figure S1. Loading scores of principle component analysis.

Additional file 5: Table S4. Summary statistics of raw data for RNA sequencing data.

Additional file 6: Table S5. Summary statistics for mapping information of RNA sequencing data.

Additional file 7: Table S6. The detailed information of DEGs.

Additional file 8: Table S7.Venn analysis of DEGs.

Additional file 9: Table S8. H-cluster (hierarchical clustering) analysis of DEGs.

Additional file 10: Table S9. Yield related genes analyses of DEGs.

Additional file 11: Table S10.GO enrichment analysis of DEGs.

Additional file 12: Table S11. KEGG enrichment analysis of DEGs.


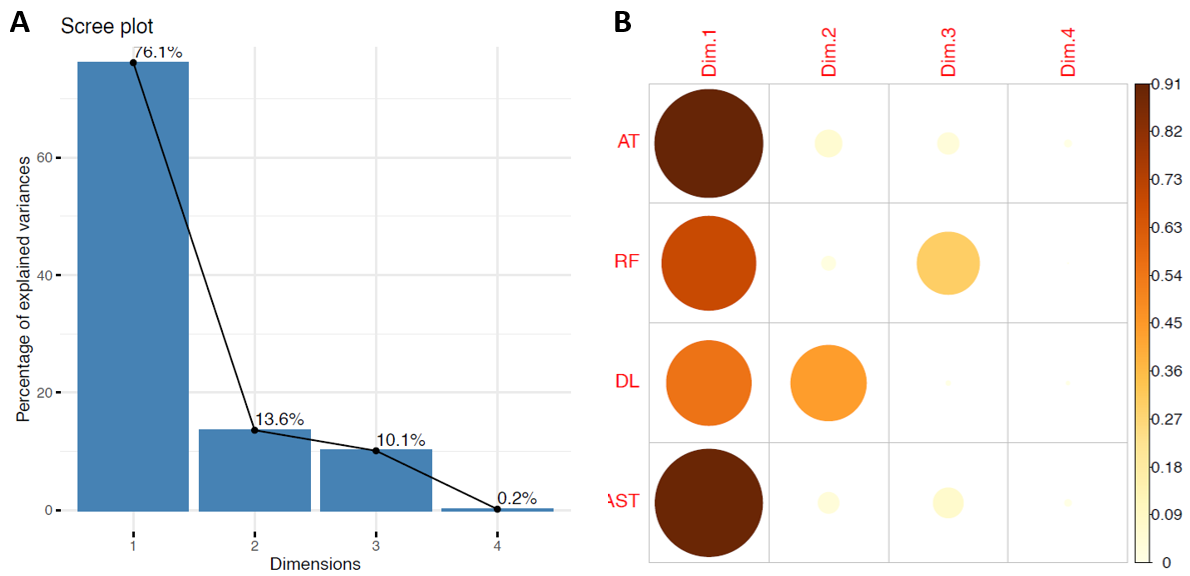


**Additional file 4: Supplementary Figure 1.** Loading scores of principle component analysis. (A) PCA percent of variance explained by PC1 through PC4. Most variance explained by the first and second PC components (total 89.7%, 76.1% + 13.6%). (B) Relative weighting of the phenotypic variables. AT, average temperature; RF, rainfall; DL, daylight length; AST, average soil temperature of 20 cm.
